# Effects of Mindset and Dietary Restraint on Attention Bias for Food and Food Intake

**DOI:** 10.5334/joc.236

**Published:** 2022-08-11

**Authors:** Sarah Kochs, Leonardo Pimpini, Wieske van Zoest, Anita Jansen, Anne Roefs

**Affiliations:** 1 Department of Clinical Psychological Science, Faculty of Psychology and Neuroscience, Maastricht University, P.O. Box 616 6200 MD Maastricht, The Netherlands; 2 School of Psychology, University of Birmingham, Edgbaston, Birmingham, United Kingdom

**Keywords:** mindset, dietary restraint, attentional bias, food intake, eye-tracking, bogus taste test

## Abstract

Evidence for attention bias (AB) for food in restrained eaters is inconsistent. A person’s mindset related to food – that is, whether someone focuses on the hedonic or health aspects of food – might be an overlooked influence on AB for food, possibly explaining the inconsistency in the literature. Fluctuations between a hedonic versus a health mindset might be strongest in restrained eaters, who have a conflicted relationship with food. We investigated the effect of mindset and dietary restraint on AB for food and food intake. We hypothesized that AB for food, as reflected in eye-movement measures and manual response latencies, as well as food intake, would be larger in the hedonic than in the health mindset, most strongly in participants scoring high on dietary restraint. Moreover, we expected a positive correlation between AB for food and food intake, especially in the hedonic mindset. We used short video clips to induce either a health or hedonic mindset. Subsequently, participants (*n* = 122) performed a modified additional singleton task with pictures of high-caloric food vs neutral pictures as irrelevant distractors. Next, food intake was measured in a bogus taste test. We found no evidence for an AB towards food, nor any moderation by either mindset or dietary restraint. Food intake tended to be higher for participants scoring higher on dietary restraint, but effects were not moderated by mindset. Response-latency based AB for food tended to correlate positively with food intake in the hedonic mindset. Taken together, our hypotheses regarding AB for food were largely not confirmed. We provide suggestions on how to improve upon the specific implementations of our AB task and mindset manipulation, to strengthen future research in this field.

## Introduction

The prevalence of overweight and obesity has reached an epidemic scope (Berghofer et al., 2008; Flegal, Carroll, Ogden, & Curtin, 2010; WHO, 2020), which is a cause for concern because overweight and obesity are associated with harmful health outcomes and high health care costs (Finkelstein, Ruhm, & Kosa, 2005; WHO, 2020). Today’s obesogenic food environment, in which cheap and easily obtainable high-caloric food is omnipresent and heavily advertised, likely plays a role in the development and maintenance of the high prevalence of overweight and obesity (Hill & Peters, 1998; Morland & Evenson, 2009; Townshend & Lake, 2017). A common response to the obesogenic environment and the resulting weight gain is the development of dietary restraint, which is characterized by chronic weight concerns and dieting attempts (Herman & Polivy, 1980). However, dietary restraint is often unsuccessful and restrained eaters tend to have a higher body-mass-index (BMI) than unrestrained eaters (Jansen, 2016; Snoek, van Strien, Janssens, & Engels, 2008). Adhering to a diet is notoriously difficult, and long-term weight-loss maintenance is often poor (Fildes et al., 2015). It has been proposed that chronic dietary restraint and perceived food deprivation are associated with increased attractiveness of food and attentional bias (AB) for food (Brooks, Prince, Stahl, Campbell, & Treasure, 2011; Polivy & Herman, 2017).

AB for food denotes selective attentional processing of food stimuli and includes voluntary and involuntary attentional processes (Werthmann, Jansen, & Roefs, 2015). AB for food is proposed to be a factor in the development and maintenance of weight related problems (Meule & Platte, 2016), and has been suggested to affect food-related decisions. A recent meta-analysis showed that an AB for food is associated with craving, hunger, and food intake (Hardman et al., 2021). It has been proposed as well that AB for food is increased in restrained eaters (Polivy & Herman, 2017), but the empirical evidence for this suggestion is inconclusive (Roefs, Houben, & Werthmann, 2015; Werthmann et al., 2015). Some studies found evidence for increased attention for food in restrained eaters (Brooks et al., 2011; Dobson & Dozois, 2004; Forestell, Lau, Gyurovski, Dickter, & Haque, 2012; Hepworth, Mogg, Brignell, & Bradley, 2010; Meule, Vogele, & Kubler, 2012; Neimeijer, de Jong, & Roefs, 2013), whereas other studies found evidence of attentional avoidance of food in restrained eaters (Hotham, Sharma, & Hamilton-West, 2012), or of an approach-avoidance pattern (i.e., attentional approach combined with attentional avoidance; Hollitt, Kemps, Tiggemann, Smeets, & Mills, 2010). Notably, several studies found no significant difference in AB for food in restrained compared to unrestrained eaters (Ahern, Field, Yokum, Bohon, & Stice, 2010; Boon, Vogelzang, & Jansen, 2000; Johansson, Ghaderi, & Andersson, 2005; Werthmann et al., 2013; Wilson & Wallis, 2013). So, overall, the picture emerging from previous empirical studies is mixed. The goal of the current study is to investigate if restrained eaters may specifically have an AB for high-caloric food when they focus on food enjoyment in a hedonic mindset. It will also be explored if early and late attentional selection are differently affected by mindset.

In line with the mixed findings in the AB-literature, it has been proposed that AB for food might best be conceptualized as a situational state instead of a relatively stable person-characteristic. That is, AB for food might be reflective of someone’s current motivational state and therefore fluctuate (Papies, Stroebe, & Aarts, 2008; Roefs et al., 2015; Werthmann et al., 2015). Recently, a new method to analyze the time-series of attention bias over the course of an experimental task, trial-level bias score (TL-BS), has been introduced (Zvielli, Bernstein, & Koster, 2015). By using this method, it has been shown that AB for food fluctuates over the course of a study within participants (Liu, Roefs, Werthmann, & Nederkoorn, 2019). This attentional fluctuation, that is attention towards and away from high caloric food, might be a result of the double-sided nature of high-caloric palatable foods. On the one hand, high-caloric food has a high hedonic value because of its good taste, but on the other hand, it has a low health value, because its caloric density is associated with weight gain and negative health outcomes. People may fluctuate between focusing on hedonic and health-related aspects of high-caloric food, depending on situational and cognitive factors. That is, people may look differently at food depending on their mindset.

Mindset describes the aspects that are on the foreground of one’s mind when thinking about food (Bhanji & Beer, 2012). Mindset likely fluctuates over time and these fluctuations may depend on subtle context cues (Werthmann, Jansen, & Roefs, 2016). We will investigate effects of a health mindset, which frames food in term of health-related aspects, and of a hedonic mindset, which frames food in terms of pleasurable aspects of food consumption. Mindset may focus on any aspect of food. Mindset has been shown to affect food perception in several ways. For example, it appears that brain responses to food stimuli are influenced by mindset (Bhanji & Beer, 2012; Franssen, Jansen, van den Hurk, Roebroeck, & Roefs, 2020; Hare, Malmaud, & Rangel, 2011). When focusing on health, brain responses to health cues were increased and food choices were in favor of healthier options (Hare et al., 2011). In contrast, increased activity in the mesocorticolimbic system of the brain was observed in a hedonic attentional focus compared to a neutral attentional focus (Franssen et al., 2020). This indicates that the salience of food might depend on mindset. In addition, food intake has been shown to be influenced by mindset as well. More specifically, portion size decisions were influenced by mindset, such that smaller portions were selected in a health mindset than in a fullness mindset (Hege et al., 2018; Veit et al., 2020). Interestingly, chocolate consumption in a so-called taste test was influenced by mindset, such that participants consumed a larger amount of chocolate in a loss of control mindset compared to a control mindset (Franssen et al., 2020).

Taken together, mindset might be a crucial determinant of AB towards food. That is AB might be directed towards food in a hedonic mindset, whereas AB might be directed away from food when in a health mindset. So, an AB for food might depend on situational states rather than relatively stable person characteristics (Field et al., 2016; Roefs, Franssen, & Jansen, 2018; Roefs et al., 2015). In line with this idea, it was found that an experimentally induced health mindset reduced AB towards food in individuals with higher levels of dietary restraint (Werthmann et al., 2016).

Effects of mindset on AB for high-caloric food are likely affected by top-down factors, such as expectations, strategy and goals, and might need some time to develop, and therefore might be most pronounced in later stages of attentional processing (Roefs et al., 2015). In contrast, early stages of attention appear to be affected more by low-level non-strategic bottom-up factors, such as the physical salience of a stimulus (van Zoest, Donk, & Theeuwes, 2004) and automatic influences of reward history (Hickey & Van Zoest, 2012). Therefore, it might be beneficial to investigate early and late attentional processes separately. Analysis methods that allow for a distinction between early and late attentional processing might be most suitable to detect an effect of mindset on AB for high-caloric food.

People may not always choose their mindset deliberately, as many factors – such as culture, media, and social networks – will influence mindset (Crum & Lyddy, 2014; Crum & Zuckerman, 2017), yet mindset will influence cognition and behavior (Crum, Salovey, & Achor, 2013). It is conceivable that, especially in a hedonic mindset, food cues in the environment attract attention even when one does not have explicit eating intentions. Effects of an AB for food might be most detrimental when people have no explicit eating intentions. For example, when people are in a hedonic mindset, a chocolate advertisement on a website during a work-related web search might capture attention and trigger the urge to consume chocolate. Food consumption in such situations may lead to problematic weight gain as it is likely driven by hedonic factors rather than physiological needs. If a researcher wants to assess this type of attentional capture, a paradigm is needed in which food does not share critical features with core components required for task performance (Cunningham & Egeth, 2018). However, most previous studies on AB for food used tasks in which food is a centrally presented and therefore difficult to ignore, such as the modified Stroop task or the visual probe task (Field et al., 2016; Roefs et al., 2015; Werthmann et al., 2015). The effects observed in these studies therefore may not be ecologically valid.

Importantly, it has been shown that an entirely task-irrelevant stimulus can capture attention (Cunningham & Egeth, 2018; Forster & Lavie, 2011). That is, a stimulus that does not share any critical features with a response target could still interfere with task performance. Therefore, using an experimental paradigm in which food items are completely irrelevant for correct task performance might be most informative and ecologically valid. The current study employed a modified version of the additional singleton task (Theeuwes, Kramer, Hahn, & Irwin, 1998), which is a type of visual search task. In this task, participants need to locate and identify a neutral target stimulus presented alongside neutral filler stimuli, while a picture of a high-caloric food or a neutral item suddenly appears as a distractor. Importantly, pictures of high-caloric food or neutral pictures are completely irrelevant for correct task performance, and participants are instructed to ignore them. In this way, the current task might resemble everyday situations, in which an AB towards food might be most detrimental, more closely than tasks in which food items are a core element for task completion.

The current study aimed to assess the effect of mindset and dietary restraint on AB for high-caloric food. Therefore, we manipulated mindset to be focused on either hedonic or health-related aspects of food. We hypothesized that AB for high-caloric food, as reflected in eye-movements and manual response latencies, would be larger in a hedonic mindset than in a health mindset, most strongly in participants scoring high on dietary restraint. For our exploratory analysis, we presumed that effects of mindset were based on late top-down attention components (Roefs et al., 2015), which are observable on eye-movements (saccades) with a long onset latency (van Zoest et al., 2004; van Zoest, Hunt, & Kingstone, 2010). To assess late attention components, we grouped trials based on saccade onset latency. We expected that effects of mindset would be more pronounced on trials with slow saccade onset compared to trials with fast saccade onset.

Additionally, we were interested in the effects of mindset and dietary restraint on intake of high-caloric food, as measured in a bogus taste test. We expected that participants would consume more high-caloric food in the hedonic mindset compared to the health mindset, and that this pattern would be more pronounced in participants with high levels of dietary restraint. Additionally, we tested the hypothesis that AB for food was positively correlated with food intake, specifically in the hedonic mindset.

## Method

### Participants

A power analysis conducted in G-Power (Faul, Erdfelder, Buchner, & Lang, 2009; Faul, Erdfelder, Lang, & Buchner, 2007) indicated that, for detecting a medium effect size (f = 0.25) in an ANCOVA design (fixed effects, main effects, interactions) with α of .05 and power of .80, 128 participants were required. Participants were recruited via advertisements on university notification boards, the university’s student research participation system, and social media. Interested individuals were screened for eligibility. One hundred and twenty-three non-obese women, varying on dietary restraint, took part in the study. We only recruited women because women display a higher prevalence of dieting than men (Hill, 2002) and therefore understanding effects of dietary restraint is more relevant for women. One participant was excluded from the study due to problems with eye-tracker calibration. The final sample consisted of 122 participants (BMI: *M* = 21.71, *SD* = 2.34, range 17.59 – 27.92; age: *M* = 21.22, *SD* = 2.68, range 18 – 30; dietary restraint: *M* = 13.98, *SD* = 4.99, range 3 – 26). Each participant provided written informed consent before participating. Each participant received a gift voucher of €10 or a course credit as compensation for participating and received a debriefing after the study was entirely completed. The Ethical Committee of the Faculty of Psychology and Neuroscience of Maastricht University approved the study. The study was pre-registered on AsPredicted (https://aspredicted.org/WQN_M9P). We deviated from the pre-registered dependent variables, because our design was not well suited to analyze saccade accuracy and because we used saccade latency to create bins for the exploratory time-course analysis. To replace the dependent variables we did not analyze, we analyzed the percentage of trials with a fixation on the distractor and the duration of the first fixation on the distractor, because these variables are frequently analyzed in studies using the additional singleton paradigm (e.g., Becker, 2010; van Zoest & Donk, 2005).

### Materials

#### Questionnaires

##### Online screening

An online questionnaire was administered to exclude participants with severe underweight (BMI < 17.5) or obesity (BMI > 30), and participants with vision impairments who do not wear contact lenses. In addition, the questionnaire assessed dietary restraint, to pseudo-randomize participants to mindset conditions while stratifying for dietary restraint. The questionnaire contained all 11 questions of the revised Restraint Scale (Herman & Polivy, 1980), asked for height and weight, and inquired about eyesight. Questions of interest were intermixed with distractor items to obscure the purpose of the questionnaire. Lifestyle-related questions, such as “How many hours do you sleep per night on average?”, were used as distractor items.

##### Hunger assessment

To standardize hunger level, the participant was asked to eat a snack (such as a sandwich) two hours before participation, and to refrain from eating and drinking anything except water in the two hours preceding participation. The participant was asked to report the time of her last meal and to describe what she had eaten on that occasion. Hunger level was assessed digitally with the question: “How hungry do you feel at this moment?”, which the participant could answer on a 100 mm visual analogue scale (VAS) ranging from 0 (not hungry at all) to 100 (very hungry).

##### Awareness check

To assess awareness of the aim of the study, the participant was asked to answer the following question on a blank sheet of paper: “Please write down your thoughts and remarks about the experiment. What is the aim of the experiment according to you?”.

##### Restraint Scale

Each participant’s level of dietary restraint was determined with the revised Restraint Scale (Herman & Polivy, 1980), which includes 11 items assessing body weight concerns and dieting intentions. Note that the revised Restraint Scale measures the intention to restrict caloric intake, not actual calorie intake restriction. We used the revised Restraint Scale because we were interested in chronic on-off dieters. The minimum score of this questionnaire is 0, and the maximum is 35, with higher scores reflecting higher levels of dietary restraint. The internal consistency of the Restraint Scale in the present sample was acceptable (Cronbach’s α = 0.77).

#### Apparatus

##### Eye-tracking

Eye movements were recorded with an Eyelink 1000 tower-mount system (1000 Hz temporal resolution, 0.01° gaze resolution, a gaze position accuracy of 0.5; SR Research Ltd., Canada), which was used with a chinrest to minimize head movements. Calibration of the eye-tracking system was performed using a nine-point calibration procedure. Saccades and fixations were defined by Eyelink 1000’s online parser. An eye-position sample was considered as belonging to a saccade if its velocity exceeded 30°/sec or its acceleration exceeded 8000°/sec/sec.

##### Stimulus presentation

Stimuli were presented on a 32-inch monitor (Philips) with a resolution of 1920 × 1080 pixels and a refresh rate of 100 Hz. The participant was seated at a distance of 57 cm from the screen, such that 1° visual angle corresponded to approximately 1 cm.

#### Mindset manipulation videos

Participants were pseudo-randomly assigned to either the health mindset or hedonic mindset. The mindsets were induced by means of short video clips (of approx. 80 s duration). The clip used to induce a health mindset displayed images and short scenes of people exercising, and pictured healthy food, such as fruit bowls and salads. Short written messages such as ‘be active’ or ‘healthy choice’ were superimposed on the images. The clip used to induce the hedonic mindset depicted images and short scenes of high-caloric food, presented in an appealing manner, and showed people enjoying food together. Short written messages such as ‘have a good time’ or ‘indulge’ were superimposed on the images. Both clips were accompanied by mindset-matching instrumental music, and the participant was asked to listen to it via headphones, to increase immersion into the mindset.

Effectiveness of the mindset manipulation was assessed with six manipulation check questions, which the participant answered on 100 mm VAS. The questions were: “To what extent were you able to immerse yourself into the video clip? very low extent – very high extent” (1: *Immersion*), “How is your current mood? very good – very bad” (2: *Mood*), “How important is enjoying food to you at this moment? not important at all – very important” (3: *Enjoyment*), “How much would you like to indulge in tasty food at this moment? not at all – very much” (4: *Indulge*), “How important is health to you at this moment? not important at all – very important” (5: *Health*), “How inclined are you to choose healthy food at this moment? not inclined at all – very inclined” (6: *Healthy choice*). The questions were presented in pseudo-random order, with the first two questions always appearing first in fixed order, as these were control questions, and the remaining four questions appearing in an individualized random order.

In addition, the effectiveness of the mindset manipulation videos was tested beforehand in a pilot study in an independent sample of participants (*n* = 23). In this pilot study, the manipulation appeared to work as intended (see Appendix Table 4 for results of the pilot study). Participants in the hedonic mindset (*M* = 7.07, *SD* = 1.74) tended to rate the importance of enjoyment higher than participants in the health mindset (*M* = 5.83, *SD* = 1.48; *t*(21) = 1.825, *p* = .082, *d* = 0.765). Participants in the health mindset (*M* = 7.59, *SD* = 1.21) tended to rate the importance of health higher than participants in the hedonic mindset (*M* = 5.60, *SD* = 3.00; *t*(14.731) = 2.124, *p* = .051, *d* = 0.872).

#### Additional Singleton Task

##### Trial and block descriptions

**Figure 1:**
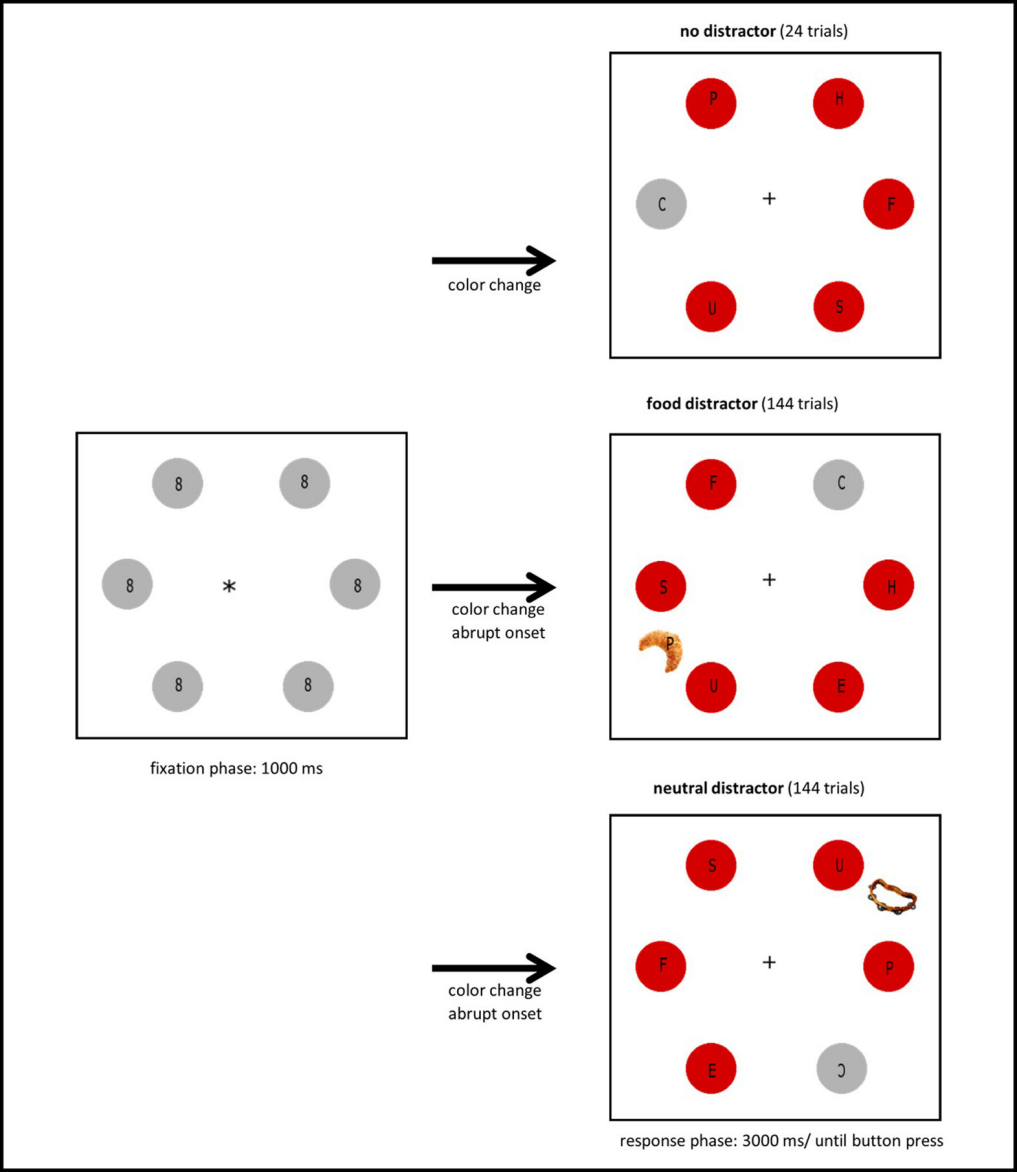
Modified additional singleton task; each participant performed 312 trials of this task.

A modified version of the additional singleton paradigm (Theeuwes et al., 1998) was used (Figure 1). The initial display was composed of six grey circles (3.7° in diameter), which were placed equally spaced (appearing on clock positions: 1, 3, 5, 7, 9, 11) on an imaginary circle with a radius of 12.6°. The six circles contained small figure-eight masks (0.2° × 0.4°). A small black fixation cross (RGB: 0 0 0, 0.4°) was presented in the middle of the imaginary circle. The display was presented for 1000 ms and the participant was instructed to fixate her gaze at the central fixation cross. After 1000 ms, circles changed color, such that five circles turned red, and one circle remained grey (=the target). The masks inside the circles that turned red changed to small letters (E, F, H, P, S, or U), and to C or reverse C in the circle that remained grey (=target). The participant was instructed to make a saccade to the target circle as soon as the color change happened and to indicate if the letter in target circle was a C or reversed C via a press on a button box. In approximately 90 percent of the trials (i.e., on 288 trials), a distractor item was added to display at the time of the color change, placed on the imaginary circle at a separation of either 90° or 150° from the target circle. The distractor was a high-caloric food item half of the time (i.e., on 144 trials), and a musical instrument the other half of the time (i.e., on 144 trials). The distractor item also contained one of the small letters. The participant was told to ignore the distractor. The task was performed in two blocks of 156 trials each for a total of 312 trials. A blank screen (duration: 500 ms) was presented in between trials. The two blocks of interest were preceded by one practice block consisting of 30 trials. In ten percent of the practice trials, a red circle was used as distractor. In the other practice trials, no distractor was added to the display.

##### Distractor items

Seventy high resolution (96 pixels/inch) color pictures were used as distractor stimuli. Thirty-five images depicted musical instruments (neutral distractors), and 35 images depicted high-caloric food items. The displayed objects were presented on a transparent background. Stimuli had an original size of 454 × 454 pixels and were presented at size of 3.7° of visual angle. Stimuli were retrieved from the internet and from the database of the Eating Behavior Laboratory of Salzburg University (Blechert, Lender, Polk, Busch, & Ohla, 2019; Blechert, Meule, Busch, & Ohla, 2014).

#### Bogus taste test

The participant was presented with four different types of high-caloric snack foods: salted (5.51 kcal/g) and paprika flavored (5.38 kcal/g) crisps (brand: Lay’s), M&M’s (5.12 kcal/g), and Maltesers (5.00 kcal/g). The foods were placed in four glass bowls (Ø 20.5 cm for crisps, Ø 13.5 cm for chocolates), which were filled generously, such that consumption of a moderate amount of food would not be easily noticeable. Bowls contained on average respectively 562.27 g of salted crisps, 572.34 g of paprika flavored crisps, 913.55 g of M&M’s, and 639.77 g of Maltesers. Questionnaires to assess taste perception on 100 mm VAS rating scales were placed with each bowl (e.g., “How tasty do you find the crisps?”; see Appendix Table 3 for the specific questions). The participant was instructed to taste and rate the food and was given exactly ten minutes. She was told that if she was finished before this time had passed, she could taste some more of the food but was asked to not change answers on the taste perception questionnaire anymore. Unbeknownst to participants, the foods were weighed (with a precision balance PB3002 Mettler Toledo) before and after the taste test to calculate the total number of calories consumed.

#### Procedure

The participant was screened with an online questionnaire approximately one week before participation. At the beginning of the scheduled session, the participant was welcomed, received information about the experiment, and signed an informed consent form. Then, the participant received instructions about the additional singleton task, was seated in front of the stimulus presentation monitor, and was asked to place her head on the chinrest. Thereafter, the participant filled in the hunger assessment questionnaire. Then, the eye-tracker was calibrated. Subsequently, the practice block of the additional singleton task was performed. After the practice block, the mindset manipulation video clip was played, and the participant answered the manipulation check questions. Then, the eye-tracker was calibrated again, and this was followed by the first block of the additional singleton task. After the first block, the mindset manipulation video and the manipulation check questions were repeated to boost the manipulation. The eye-tracker was calibrated again, and the second block of the additional singleton paradigm was performed. Next, the participant was accompanied to another room for the bogus taste test, and afterwards she filled in the questionnaire on awareness of the study’s aim and completed the revised restraint scale.^1^ Next, height and weight of the participant were measured. Finally, the participant was thanked and received compensation for participating.

#### Analyses

##### Manipulation check

The responses to each question of the manipulation check were averaged across the measurement after the first and the second presentation of the mindset manipulation moments. The score on each question was analyzed in an ANCOVA with mindset (health vs. hedonic) as fixed factor and dietary restraint as covariate (mean-centered).

##### Additional singleton task

Data were preprocessed and prepared for the main statistical analyses as follows: Information about saccades and fixations were extracted from the eye-tracking data files. Interest areas around the fixation cross (1.5° in size), target (6° in size), and distractor (6° in size) were defined. Saccades and fixations were to be considered on the object if they fell into the corresponding interest area. Trials were excluded based on the following exclusion criteria: For the eye-tracking measures, trials with first saccade onset faster than 80 ms (0.926%) or slower than 600 ms (0.899%) were excluded from the analyses (van Zoest & Donk, 2008; van Zoest et al., 2004). In addition, trials with the first saccade not starting from within 1.5° around the fixation cross (4.517%) were excluded from the analysis. Those criteria led to a total exclusion of 5.547% of the trials. For manual response latency analyses, these trials were also excluded. In addition, trials without button press (0.055%) or with wrong button press (2.731%) were excluded from manual response latency analysis. Also, trials with a manual response latency shorter than 100 ms (0.002%) or longer than 2000 ms (0.457%) were excluded (e.g., Theeuwes, De Vries, & Godijn, 2003). Next, trials with a manual response latency shorter than the participant’s mean – 3 *SD* (0.002%) or longer than mean + 3 *SD* (1.548%) were excluded (e.g., Castellanos et al., 2009; Mogg, Bradley, Hyare, & Lee, 1998; Werthmann et al., 2011). These criteria led to an exclusion of an additional 4.419% of the trials. Based on all exclusion criteria, 9.341% of trials were excluded from manual response latency analysis. Participants of whom more than one-third of the trials had to be excluded based on these criteria were excluded from data analyses. This led to the exclusion of five participants.

The analyses of the eye-tracking measurements focused on three main dependent variables: (1) the percentage of trials in which a fixation on the distractor occurred, (2) the duration of the first fixation on the distractor, (3) the total amount of time (i.e., dwell time) that the distractor was fixated on per trial. In addition, manual response latency and response accuracy were analyzed. Each dependent variable was analyzed in a mixed ANCOVA, with distractor type (neutral vs. food; within-subjects) and mindset (health vs. hedonic; between-subjects) as fixed factors and dietary restraint as covariate (mean-centered).

##### Time-course analysis

It is conceivable that the effect of mindset is only apparent on later attention components, as it is likely based on top-down attention processes, and that attentional selection develops over time (e.g., van Zoest et al., 2004). To test this, exploratory analyses were performed to assess if saccadic latency (i.e., onset time of the first saccade after the color change) influenced the percentage of trials with a fixation on the distractor. Therefore, for each participant, trials were grouped into three bins (thirds of the data) according to saccadic latency (fast saccade onset, medium saccade onset, slow saccade onset). The percentage of trials with a fixation on the distractor was analyzed in a mixed ANCOVA with bin (fast, medium, slow; within-subjects), distractor type (neutral vs. food; within-subjects) and mindset (health vs. hedonic; between-subjects) as fixed factors and dietary restraint as covariate (mean-centered).

##### Bogus taste test

For each of the four snack foods, the amount eaten by the participant was determined and the total number of calories consumed was calculated. Total calorie intake was analyzed in an ANCOVA with mindset (health vs. hedonic; between-subjects) as fixed factor and dietary restraint as covariate (mean-centered). Furthermore, correlations between total calorie intake and the dependent eye-tracking variables (described above) as well as manual response latency were calculated. To do so, for each dependent AB variable, a bias score was computed by subtracting the mean response of trials with a neutral distractor from the mean response of trials with a food distractor. A positive bias score reflects an AB towards food, whereas a negative bias score reflects an AB away from food. Correlations between food intake and bias scores were calculated within and across mindsets.

## Results

### Manipulation check

As expected, participants in the health and hedonic mindset did not differ in their scores on the control items *Immersion* and *Mood*. Contrary to expectations, there was no significant difference between mindsets on *Enjoyment*. As expected, participants in the hedonic mindset scored higher on *Indulge* than participants in the health mindset. In addition, participants in the health mindset scored higher on *Health* and *Healthy choice* than participants in the hedonic mindset. Scores on *Health* and *Healthy choice* were influenced by dietary restraint, such that participants with higher levels of dietary restraint scored higher on *Health* and *Healthy choice*. None of the mindset × dietary restraint interactions reached significance. See Table 1 for all relevant statistics. Overall, three of the four relevant items showed significant differences between mindsets in line with our expectations. Thus, it appears that our mindset manipulation was effective, as evidenced by medium to large effect sizes on relevant items.

**Table 1:** Mindset manipulation check results; * = trend-level significant at *p* < .10, ** = significant at *p* < .05, *** = significant at *p* < .01; *M*: mean, *SD*: standard deviation, *d*: Cohen’s d.


ITEM		*M (SD)*	MINDSET			DIETARY RESTRAINT		MINDSET × DIETARY RESTRAINT	
		
			*F*(1,118)	*p*	*d*	*F*(1,118)	*p*	*F*(1,118)	*p*

**Immersion**	health	67.84 (16.41)	0.012	.912	0.024	0.045	.832	0.929	.337

	hedonic	68.25 (17.55)							

**Mood**	health	68.60 (14.44)	0.005	.941	0.029	0.825	.365	0.102	.750

	hedonic	69.08 (18.44)							

**Enjoyment**	health	72.66 (16.61)	0.007	.932	0.026	0.347	.557	0.104	.747

	hedonic	72.20 (18.82)							

**Indulge**	health	61.10 (20.82)	4.603	.034**	0.379	0.325	.570	2.370	.126

	hedonic	69.14 (21.55)							

**Health**	health	77.89 (15.16)	5.779	.018**	0.474	8.639	.004***	0.830	.364

	hedonic	68.65 (23.07)							

**Healthy choice**	health	74.70 (15.33)	23.104	.000005*	0.887	15.022	.0002***	2.596	.110

	hedonic	57.04 (23.62)							


### Hunger check

Overall, participants reported moderate hunger levels (*M* = 42.02, *SD* = 26.42). On average, participants complied with the instruction to eat two hours before participation but not within the two preceding hours (average time since last eating occasion: *M* = 141.31 minutes, *SD* = 58.26 minutes). Hunger level did not differ significantly between mindsets (health: *M* = 41.33, *SD* = 26.94, hedonic: *M* = 42.70, *SD* = 26.09, *F*(1,118) = 0.049, *p* = .825). There was no significant effect of dietary restraint on hunger level (*F*(1,118) = 0.459, *p* = .500), and no significant interaction between dietary restraint and mindset (*F*(1,118) = 2.461, *p* = .119).

### Dependent variables eye-tracking

#### Percentage of trials with fixation on distractor

Unexpectedly, overall, the neutral distractor tended to be fixated on a slightly higher percentage of trials than the food distractor (*F*(1,113) = 2.771, *p* = .099, η_p_^2^ = 0.024; Table 2). In addition, the distractor – independent of distractor *type* – was fixated on a greater percentage of trials in the hedonic mindset than in the health mindset (*F*(1,113) = 3.992, *p* = .048, η_p_^2^ = 0.034). The mindset × dietary restraint interaction was significant (*F*(1,113) = 4.208, *p* = .043, η_p_^2^ = 0.036) as well. Splitting the sample in restrained eaters (scoring 15 and higher on revised Restraint Scale, *n* = 52) and unrestrained eaters (scoring 14 or lower on the revised Restraint Scale, *n* = 65) showed that for unrestrained eaters the percentage of trials with a fixation on the distractor was higher in the hedonic mindset (*M* = 16.06, *SD* = 14.64) than in the health mindset (*M* = 10.21, *SD* = 6.95; *t*(43.999) = 2.049, *p* = .046, *d* = 0.511), but did not differ significantly for restrained eaters (health: *M* = 9.94, *SD* = 6.53; hedonic: *M* = 11.02, *SD* = 7.44; *t*(50) = 0.557, *p* = .58, *d* = 0.154). No other effects reached significance, all *F*(1,113) < 2.377, all *p* > .126. See Table 2 for an overview of the statistics.

**Table 2:** Overview of effects from analysis of additional singleton task; * = trend-level significant at *p* < .10, ** = significant at *p* < .05; *M*: mean, *SD*: standard deviation.


DESCRIPTIVE STATISTICS	PERCENTAGE OF TRIALS WITH FIXATION ON DISTRACTOR		DURATION OF FIRST FIXATION ON DISTRACTOR		DWELL TIME ON DISTRACTOR		MANUAL RESPONSE LATENCY		RESPONSE ACCURACY	
				
	*M (SD)*		*M (SD)*		*M (SD)*		*M (SD)*		*M (SD)*	
				
	NEUTRAL	FOOD	NEUTRAL	FOOD	NEUTRAL	FOOD	NEUTRAL	FOOD	NEUTRAL	FOOD

**health**	10.62 (6.67)	9.55 (7.33)	86.38 (22.86)	91.51 (28.14)	94.84 (30.11)	100.49 (40.02)	797.48 (139.96)	795.82 (145.15)	97.07 (2.55)	97.14 (2.59)

**hedonic**	14.03 (11.97)	13.78 (12.92)	84.48 (22.07)	85.22 (24.86)	92.45 (30.23)	95.23 (34.62)	803.05 (114.30)	802.01 (116.23)	97.26 (1.88)	97.51 (1.83)

**Inferential statistics**	***F***(1,113) ***(p)***		***F***(1,111) ***(p)***		***F***(1,111) ***(p)***		***F***(1,113) ***(p)***		***F***(1,113) ***(p)***	

**distractor**	2.771 (.099*)		1.610 (.207)		1.990 (.161)		0.578 (.449)		0.987 (.323)	

**mindset**	3.992 (.048**)		0.833 (.363)		0.290 (.592)		0.030 (.863)		0.651 (.421)	

**dietary restraint**	1.883 (.173)		0.649 (.422)		1.265 (.263)		0.566 (.454)		1.255 (.265)	

**distractor × mindset**	0.808 (.371)		0.734 (.393)		0.131 (.718)		0.003 (.954)		0.428 (.515)	

**distractor × dietary restraint**	2.377 (.126)		1.873 (.174)		1.840 (.178)		2.099 (.15)		0.643 (.424)	

**mindset × dietary restraint**	4.208 (.043**)		0.043 (.836)		0.305 (.582)		0.032 (.859)		0.000009 (.998)	

**distractor × mindset × dietary restraint**	0.049 (.825)		0.203 (.654)		0.578 (.449)		0.590 (.444)		0.120 (.730)	


Two participants in the hedonic mindset had a high percentage of fixations on the distractor (> *M* + 3 *SD*). When removing these participants from the analysis, the neutral distractor still tended to be fixated on a higher percentage of trials than the food distractor (*F*(1,111) = 3.845, *p* = .052, η_p_^2^ = 0.033). The main effect of mindset (*F*(1,111) = 2.036, *p* = .156, η_p_^2^ = 0.018) and the mindset × dietary restraint interaction (*F*(1,111) = 2.314, *p* = .131, η_p_^2^ = 0.020) were no longer significant. No other effects were significant after removing these two participants, all *F*(1,113) < 2.429, all *p* > .122.

#### First fixation duration and dwell time on distractor

No significant effects on the duration of the first fixation on the distractor were detected, all *F*(1,111) < 1.873, all *p* > .174. Similarly, no significant effects on the dwell time on the distractor were observed, all *F*(1,111) < 2.086, all *p* > .151. See Table 2 for an overview of the statistics. Overall, the results on the eye-tracking dependent variables were not in line with our hypotheses, as we observed no attentional bias for food, and no moderation by either dietary restraint or mindset.

### Manual response latency

No significant effects on manual response latency were found, all *F*(1,113) < 2.099, all *p* > .15. See Table 2 for an overview of the statistics. The results on manual response latency were not in line with our hypotheses, as we observed no attentional bias for food, and no moderation by either dietary restraint or mindset.

### Response accuracy

As expected, response accuracy did not differ significantly between conditions, all *F*(1,113) < 1.255, all *p* > .265. See Table 2 for an overview of the statistics.

### Time-course analyses

#### Percentage of trials with fixation on distractor

**Figure 2:**
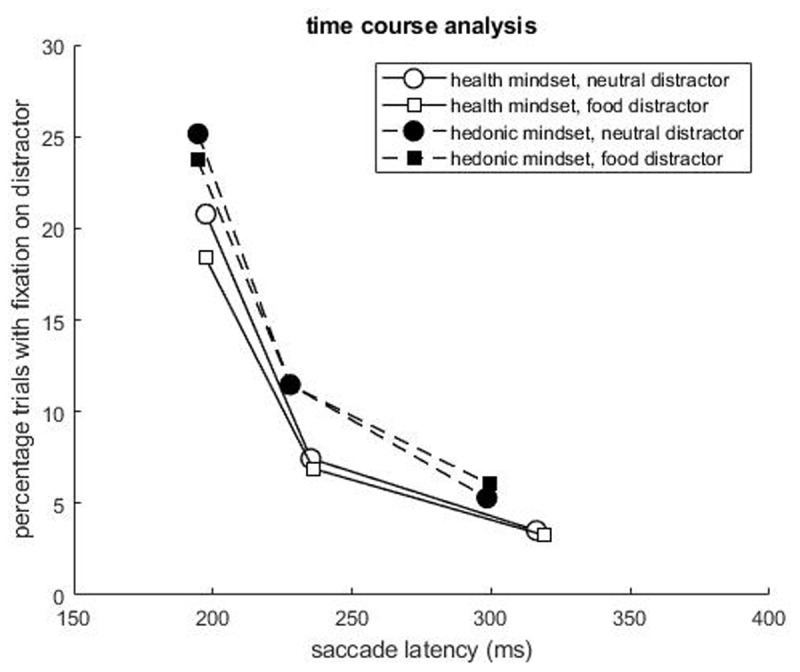
Results of the time course analysis depicting the percentage of trials with a fixation on the distractor per saccade latency bin.

We analyzed the percentage of trials with a fixation on the distractor as a function of saccade latency (grouped in 3 bins: slow, medium, fast) to explore effects of mindset on attentional selection. The percentage of trials with a fixation on the distractor differed significantly across bins (*F*(1.278,144.389) = 155.739, *p* < .001, η_p_^2^ = 0.580),^2^ with lower percentages with increasing bin. We also observed a significant interaction between bin and distractor type (*F*(1.806,204.069) = 3.525, *p* = .036, η_p_^2^ = 0.030). Against our expectations, in bin 1 (fast), the neutral distractor (*M* = 22.87, *SD* = 17.09) was fixated on a higher percentage of trials than the food distractor (*M* = 20.94, *SD* = 16.02; *t*(116) = 2.586, *p* = .011, *d* = 0.116). There was no significant difference in the percentage of trials with a fixation on the distractor between neutral and food distractors in bin 2 (medium; neutral: *M* = 9.37, *SD* = 9.93; food: *M* = 9.07, *SD* = 11.53; *t*(116) = 0.502, *p* = .616, *d* = 0.028) and bin 3 (slow; neutral: *M* = 4.37, *SD* = 6.78; food: *M* = 4.59, *SD* = 7.54; *t*(116) = 0.522, *p* = .602, *d* = 0.031). Furthermore, as in the main analysis, participants in the hedonic mindset (*M* = 13.85, *SD* = 12.22) fixated the distractor – independent of distractor *type* – on a higher percentage of trials than participants in the health mindset (*M* = 10.04, *SD* = 6.68; *F*(1,113) = 4.002, *p* = .048, η_p_^2^ = 0.034). Also, as in the main analyses, the mindset × dietary restraint interaction was significant (*F*(1,113) = 4.212, *p* = .042, η_p_^2^ = 0.036). No other effects reached significance, all *F* < 2.693, *p* >.104. See Figure 2 for the pattern of results.

After removing two participants with a rather high percentage of fixations on the distractor (> *M* + 3 *SD*), we still observed significant differences in percentage of trials with a fixation on the distractor between bins (*F*(1.223,135.727) = 153.099, *p* < .001, η_p_^2^ = 0.580). Also the interaction between bin and distractor type remained trend-level significant (*F*(1.799,199.692) = 3.074, *p* = .054, η_p_^2^ = 0.027). Also, participants tended to fixate more often on the neutral compared to the food distractor (*F*(1,111) = 3.767, *p* = .055, η_p_^2^ = 0.033). No other effects were significant after removing the two participants, all *F* < 2.41, all *p* > .123.

### Bogus taste test

We observed a trend-level effect of dietary restraint on food intake during the taste test (*F*(1,113) = 3.068, *p* = .083, η_p_^2^ = 0.026), reflecting increased food intake with increased dietary restraint. Contrary to our hypothesis, food intake during the taste test did not differ significantly between mindsets (health: *M* = 288.46 kcal, *SD* = 157.1; hedonic: *M* = 265.33, *SD* = 150.81 kcal; *F*(1,113) = 0.424, *p* = .516, η_p_^2^ = 0.004), and the dietary restraint × mindset interaction was not significant (*F*(1,113) = 1.479, *p* = .226, η_p_^2^ = 0.013).

#### Correlations AB scores with food intake

Across mindsets, no significant correlations between AB scores and food intake were observed, all *r* < .089, all *p* > .353. In the health mindset, no significant correlations between AB scores and food intake were observed either, all *r* < .157, all *p* > .243. In the hedonic mindset, we observed a trend-significant correlation between the percentage of fixation on the distractor bias score and food intake (*r*(54) = .231, *p* = .093), indicating that this AB towards food tended to be positively associated with a higher food intake during the taste test. However, when removing two participants with a rather high percentage of fixations on the distractor (> *M* + 3 *SD*), the correlation was no longer significant (*r*(52) = .174, *p* = .218). We also observed a trend-level correlation between manual response latency bias and food intake (*r*(54) = .245, *p* = .075), indicating that this AB towards food tended to be positively associated with a higher food intake during the taste test as well. Other AB scores (i.e., first fixation duration bias score, dwell time bias score) were not significantly correlated with food intake (range *r* –.050 – –.015, all *p* > .722).

#### Correlations mindset manipulation scores with food intake

Across mindsets, scores on *Immersion* tended to correlate positively with food intake during the taste test (*r*(117) = .175, *p* = .059). Scores on *Enjoyment* correlated significantly positively with food intake (*r*(117) = .184, *p* = .047), indicating that participants scoring higher on *Enjoyment* consumed more food. Similarly, scores on *Indulge* correlated significantly positively with food intake during the taste test (*r*(117) = .238, *p* = .010), indicating that participants scoring higher on *Indulge* consumed more food. No other correlations across mindsets reached significance, range *r*(117) –.100 – .055, all *p* > .282.

In the health mindset, we observed a significant correlation between scores on *Indulge* and food intake (*r*(58) = .339, *p* = .009), indicating that participants with higher scores on *Indulge* consumed more food. We also observed a marginally significant negative correlation between scores on *Health* and food intake (*r*(58) = –.222, *p* = .093), indicating that participants with higher scores on *Health* tended to consume less food. No other correlation reached significance in the health mindset, all *r*(58) < .215, all *p* > .105.

In the hedonic mindset, we observed a significant correlation between scores on *Immersion* and food intake (*r*(59) = .282, *p* = .031). We also observed a significant correlation between scores on *Enjoymen*t and food intake (*r*(59) = .307, *p* = .018), indicating that participants with higher scores on *Enjoyment* consumed more food. No other correlations reached significance in the hedonic mindset, range *r*(59) –.069 – .179, all *p* > .174.

## Discussion

The current study tested the hypothesis that participants would display a larger attention bias for high-caloric food and consume more food in the hedonic mindset than in the health mindset, most strongly in participants scoring high on dietary restraint. In addition, we explored if effects of mindset on AB for food are more pronounced on trials with a slow saccade onset. Finally, we expected that AB for food would correlate positively with food intake in the bogus taste test, especially in the hedonic mindset. The main findings include: First, the results showed no evidence for AB for food. Second, we observed no significant effect of mindset or dietary restraint on AB for food. Third, whereas mindset did not significantly affect food intake, participants scoring higher on dietary restraint tended to consume more high-caloric food during the bogus taste test. Fourth, in the hedonic mindset, manual response-latency based AB for food (but not other indicators of AB for food) tended to correlate positively with food intake during the bogus taste test.

Contrary to our hypothesis, we did not observe an AB for food at all in the present experiment. Overall, participants’ attention was captured by the irrelevant distractor (food and neutral) on a small percentage of trials only (on average on 11.91% trials). Other studies have similarly reported lack of evidence for AB for food. For example, in a Posner cueing task, no AB for food was found (Soetens, Braet, & Bosmans, 2008). Also, when investigating AB for food in overweight vs. lean individuals with a modified additional singleton task, no AB for food was observed on eye-tracking measures (Pimpini, Kochs, van Zoest, Jansen, & Roefs, 2022). In contrast to studies that failed to find evidence for a bias for food, studies that have reported an overall AB towards food typically used the dot-probe task to measure AB for food (Werthmann et al., 2015). However, recent work using an online version of dot-probe task also failed to observe an AB for food (Liu, Roefs, & Nederkoorn, 2021). One explanation why the current study failed to observe an AB for food may be because the task was too easy, due to the ratio of distractor present vs. distractor absent trials (90% vs. 10% of trials), which might have benefited the ability to overcome distraction (e.g., Sayim, Grubert, Herzog, & Krummenacher, 2010). Moreover, the distractor was highly distinct from the remainder of the stimulus display, including the target (Gaspelin, Leonard, & Luck, 2017; Poiese, Spalek, & Di Lollo, 2008), also making the task potentially too easy.

Unexpectedly, mindset did not significantly affect AB for food or food intake. Perhaps, the current mindset manipulation was not sufficient to affect AB for food because it was not directly relevant for task completion and the participant was not actively involved in creating the mindset. In another study a non-task-based passive mindset manipulation has also been (partly) ineffective (Franssen et al., 2022; Pimpini et al., 2022). Note that in some previous studies (Roefs et al., 2006; Werthmann et al., 2016) a non-task-based mindset manipulation was effective, which might be because the participant had an active role in the manipulation (e.g., devising a healthy menu). However, most previous studies that have reported effects of mindset on cognitive variables (Bhanji & Beer, 2012; Franssen et al., 2020) used a mindset manipulation that was part of the experimental task. In these studies, participants were required to evaluate food stimuli throughout the task based on either hedonic or health aspects of the food stimuli to induce a mindset. So, we most likely were not able to observe effects of mindset on AB for food – if at all present – due to the passive non-task-based mindset manipulation.

The present study revealed no significant effect of dietary restraint on AB for food. This finding is in line with previous studies that observed no effect of dietary restraint on AB for food (Ahern, Field, Yokum, Bohon, & Stice, 2010; Boon, Vogelzang, & Jansen, 2000; Johansson, Ghaderi, & Andersson, 2005; Werthmann et al., 2013; Wilson & Wallis, 2013), but contradicts studies that found evidence for an effect of dietary restraint on AB for food (Forestell, Lau, Gyurovski, Dickter, & Haque, 2012; Hepworth, Mogg, Brignell, & Bradley, 2010; Meule, Vogele, & Kubler, 2012; Neimeijer, de Jong, & Roefs, 2013). Based on previous literature (Werthmann et al., 2016), we did expect to observe an interaction between mindset and dietary restraint on AB for food. We expected that AB for food would be increased in the hedonic mindset particularly in restrained eaters. It is likely that we were unable to observe the hypothesized interaction because our mindset manipulation was not task-based and did not actively involve the participant. A more involving mindset manipulation might help to resolve the unclarity. In addition, some suboptimal parameters of the paradigm used to assess AB for food might have contributed to the lack of effect.

It is to be noted that unrestrained eaters fixated the distractor, independent of whether the distractor was a food or neutral item, more often in the hedonic than in the health mindset, whereas we observed no significant difference in percentage of fixations on the distractor between mindsets in restrained eaters.^3^ This suggests that in the current task, in which the distractor was a high-caloric food item half of the time, being in a hedonic mindset might have generally increased distractibility in unrestrained eaters. Recently, increased distractibility in a hedonic mindset compared to a health mindset was also observed in individuals with obesity (Pimpini et al., 2022). Thus, mindset could affect attentional settings more generally rather than specifically affecting AB for food.

Participants scoring high on dietary restraint tended to consume more food during the bogus taste test. This finding is surprising, especially considering some previous studies showing that restrained eaters consumed *less* food than unrestrained eaters during taste tests when no pre-load (such as a high-caloric milkshake) was given. However, other studies have shown that consumption of an actual pre-load is not always necessary for restrained eaters to feel disinhibited and increase their food intake. Food cues, such as the smell of food, appear to be sufficient to trigger increased food intake (Fedoroff, Polivy, & Herman, 2003; Jansen & Van den Hout, 1991; Polivy & Herman, 2017). In the present study, the food cues in the additional singleton task, which preceded the bogus taste test, might potentially have had a disinhibiting effect on restrained eaters and elicited increased food consumption. In addition, it has been shown that the eating behavior of restrained eaters is influenced by external cues, such as social norms (Ruderman, 1986). The test foods during the current bogus taste test were presented in very large bowls, such that a large quantity of food was available for the participants. Though this is common practice in bogus taste tests, this might have evoked the idea in restrained eaters that increased consumption is acceptable or even expected. Thus, cues in the study might have influenced restrained eaters more than unrestrained eaters to increase their food intake.

Interestingly, in the hedonic mindset we observed a positive trend-level correlation between manual response-latency based AB for food and food intake during the bogus taste test.^4^ This is in line with the results of a recent meta-analysis (Hardman et al., 2021), which detected a relation between AB for food and food intake. Thus, AB for food might be an indicator of food-related motivation and could be predictive of subsequent food intake. Interestingly, we observed the correlation between manual response-latency based AB for food and food intake only in the hedonic mindset. So, it might be that AB for food only indicates subsequent food intake when it is in line with people’s mindset. We also observed that responses to the mindset manipulation check questions were correlated with food intake during the taste test. A higher importance of food enjoyment and intention to indulge were associated with higher food intake, across as well as within mindsets, whereas higher importance of health was associated with reduced food intake, particularly in the health mindset. Also, immersion in the mindset was associated with increased food intake, especially in the hedonic mindset. This suggests that food intake might be congruent with a person’s mindset and its resulting intentions.

Though the manipulation of mindset was quite effective, as evidenced by the manipulation check, future research could improve the manipulation of mindset. Especially a manipulation that is embedded in the task to measure AB might be more effective than the current non-task-based mindset manipulation. Additionally, it might be important that the participant is actively involved in the mindset manipulation for it to have a lasting effect. In addition, some parameters of the current task have been suboptimal. This might be a reason why we have been unable to detect distracting effects of food, especially because food was irrelevant for task completion. Future research needs to improve the parameters of the additional singleton task, to test if this paradigm is suitable to study food related AB. The current results suggest that an increase of the difficulty of the task could improve the sensitivity, which could be achieved by decreasing the likelihood of distractor presence and increasing the similarity between distractors and the remainder of the search display. Overall, more research with further refined methodology is needed before conclusions considering the effect of mindset (in interaction with dietary restraint) on AB for food can be made.

## Data accessibility statement

Data and code of this study can be viewed at: https://osf.io/2bmeu/?view_only=91f24051a4404799bf8a5883ab79fe73.

## Footnotes

Dietary restraint was measured with the revised Restraint scale during the online screening and at the end of the experiment. The dietary restraint scores measured during the online screening were used for randomization purposes. The dietary restraint scores that were measured at the end of the experiment were used in the main analyses.

Results involving the factor bin are reported with Greenhouse-Geisser correction.

However, the effect was no longer significant after removing two participants with a rather high percentage of fixations on the distractor. Hence, the effect does not seem to be very robust.

However, we observed no significant correlations between other indicators of AB for food and food intake during the bogus taste test.

## Appendix

**Table 3:** Mock taste test questions: words printed in italics are placeholders for terms that differed on each form and described the actual food item to be rated.

QUESTION	ANSWER (VAS SCALE: 0 – 100 MM)

How appealing do you think the *food items* look?	not appealing at all – extremely appealing

How delicious do you think the *food items* smell?	not delicious at all – extremely delicious

How tasty do you find the *food items*?	not tasty at all – extremely tasty

How *crispy/crunchy* do you find the *food items*?	not *crispy/crunchy* at all – extremely *crispy/crunchy*

How *salty/sweet* do you find the *food items*?	not *salty/sweet* at all – extremely *salty/sweet*

How long does the taste of the *food items* stay in your mouth?	not long at all – extremely long

Which of the two types of the *food item* do you like best? (asked on every second form)	**a.** The *item* in bowl 1 **b.** The *item* in bowl 2 **c.** I do not have a preference

**Table 4:** Mindset manipulation pilot results; * = trend-level significant at *p* < .10; *M*: mean, *SD*: standard deviation.

ITEM	HEALTH *M (SD)*	HEDONIC *M (SD)*	*t*(21)	*p*

To which extent were you able to get into the spirit of the movie?	6.03 (2.04)	6.90 (1.69)	–1.11	.280

How strongly are you immersed in the movie at this moment?	5.78 (1.27)	6.02 (1.41)	–0.413	.648

How hungry do you feel right now?	4.42 (2.32)	6.20 (2.71)	–1.691	.106

How sated do you feel right now?	5.32 (2.11)	5.59 (2.25)	–0.291	.774

How important is the taste of food to you at this moment?	5.81 (2.25)	6.48 (2.35)	–0.703	.490

How important is enjoying food to you at this moment?	5.83 (1.48)	7.07 (1.74)	–1.825	.082*

How much would you like to indulge in tasty food at this moment?	4.80 (2.27)	6.49 (2.66)	–1.630	.118

How important is the calorie content of food to you at this moment?	6.18 (2.01)	4.38 (2.95)	1.771	.101

How important is health to you at this moment?	7.59 (1.21)	5.60 (3.00)	2.124	.051*

How inclined are you to choose healthy food at this moment?	7.24 (1.53)	5.47 (2.82)	1.824	.080*
